# Electrolyte‐Gated Transistor Array (20 × 20) with Low‐Programming Interference Based on Coplanar Gate Structure for Unsupervised Learning

**DOI:** 10.1002/smsc.202300306

**Published:** 2024-03-03

**Authors:** Wenkui Zhang, Jun Li, Mengjiao Li, Yi Li, Hong Lian, Wenqing Gao, Benxiao Sun, Fei Wang, Lian Cheng, Hanqi Yu, Lianghao Chen, Jianhua Zhang

**Affiliations:** ^1^ School of Microelectronics Shanghai University Shanghai 201800 China; ^2^ MOE Key Laboratory of Advanced Display and System Applications Ministry of Education Shanghai University Shanghai 200072 China

**Keywords:** arrays, electrolyte‐gated transistors, low energy consumption, unsupervised learning, winner‐takes‐all neural network

## Abstract

Compute‐in‐memory (CIM) is a pioneering approach using parallel data processing to eliminate traditional data transmission bottlenecks for faster, energy‐efficient data handling. Crossbar arrays with two‐terminal devices such as memristors and phase‐change memory are commonly employed in CIM, but they encounter challenges such as leakage current and increased power usage. Three‐terminal transistor arrays have potential solutions, yet large‐scale electrolyte‐gated transistors (EGTs) demonstrations are uncommon due to compatibility issues with existing photolithography processes. Herein, a 20 × 20 EGTs array is designed using indium‐gallium‐zinc‐oxide as the semiconductor channel and polyacrylonitrile (PAN) doped with C_2_F_6_LiNO_4_S_2_ as the electrolyte. Each transistor unit in the array can serve as a synapse, exhibiting a large conductance range, low energy consumption (6.984 fJ) for read–write operations, excellent repeatability, and quasilinear update characteristics. It has been confirmed that the EGTs array not only enables precise device programming but also virtually eliminates signal interference between neighboring devices during the programming process. Using 54 transistors in the EGTs array, unsupervised learning with a winner‐takes‐all neural network is successfully demonstrated. After 50 training iterations, the neural network achieves perfect 100% accuracy in classifying test‐set letters. The work demonstrates the potential of EGTs for constructing large‐scale integration synaptic array toward efficient computing architectures.

## Graphical abstract

Crafted with photolithography, a 20 × 20 electrolyte transistor array excels in synapse performance, with low energy consumption at 6.984 fJ and good uniformity. The designed array enables precise programming of target devices while minimizing interference among adjacent devices to almost zero. With 54 transistors, successful unsupervised learning demonstrations achieve 100% classification accuracy.

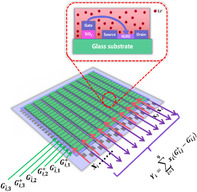

## 1 Introduction

Compute‐in‐memory (CIM), as an innovative computing paradigm, has garnered significant attention and research interest.^[^ 1, 2, 3 ^]^ It integrates computing and storage cell in parallel, effectively eliminating the need for frequent data transformation between memory and processing units in the von Neumann architecture, holding immense potential for a wide range of applications.^[^ 4, 5, 6, 7, 8 ^]^ In CIM, parallel data processing can be achieved through vector–matrix multiplication using Ohm's law (for multiplication) and Kirchhoff's law (for accumulation) in a crossbar array structure.^[^ 9, 10, 11 ^]^ Synaptic devices are employed to accurately copy the synaptic plasticity in the biological brain, underlying the precise vector–matrix multiplication. Two‐ terminal devices, such as memristors^[^ 12, 13, 14, 15 ^]^ and phase‐change memory,^[^ 15, 16, 17 ^]^ have been frequently used as synaptic devices. However, hardware arrays consisting of these two‐terminal devices to implement CIM encounter challenges in cross‐talk induced, sneak path current, and nonlinear current–voltage characteristics.^[^ 18, 19, 20 ^]^

Three‐terminal devices possess the potential to precisely modulate the conductivity of individual devices in an array, owing to their extra terminals for programming and reading.^[^ ^21^ ^]^ Among these three‐terminal devices, electrolyte‐gated transistors (EGTs), with their large range of conductivity, low read–write energy consumption, and fast switching speed, have emerged as ideal candidates for emulating biological synapses.^[^ 2, 22, 23 ^]^ However, current research on EGTs primarily focuses on enhancing the performance of individual transistors, such as reducing energy consumption,^[^ ^24^ ^]^ increasing tensile strength,^[^ ^25^ ^]^ and improving proton mobility.^[^ ^26^ ^]^ There is less emphasis on the fabrication of large‐scale, high‐density arrays of EGTs, which are essential for the implementation of integrated applications.^[^ 27, 28 ^]^ Many of the reported EGTs currently use organic or liquid electrolytes, posing significant challenges for manufacturing methods predominantly based on photolithography.^[^ 29, 30, 31 ^]^ For instance, photolithography can damage organic solid‐state electrolytes. Although EGTs employing inorganic electrolyte materials demonstrate potential for large‐area fabrication,^[^ 32, 33 ^]^ they often require higher energy consumption. Therefore, exploring alternative manufacturing methods, using organic solid‐state electrolytes as insulating layers to produce large‐scale EGTs arrays, becomes crucial. In practical arrays of three‐terminal devices, while individual devices possess the capability for precise control, a common gate electrode is often shared by a row or column of devices. In such scenarios, programming one device can often lead to interference with adjacent devices.^[^ ^34^ ^]^ To achieve precise control of devices within the array without causing programming interference to neighboring devices, a programming methodology tailored for EGTs arrays is required.

Unsupervised learning is a learning paradigm within the field of machine learning that, in contrast to supervised learning, does not require labeled target outputs, making it more adaptable to large‐scale datasets. Furthermore, it aids in the discovery of latent structures, patterns, and features within data without prior knowledge of these details, thus holding significant value in data exploration, feature selection, and data understanding.^[^ 35, 36, 37, 38, 39 ^]^ In the context of unsupervised learning applications based on synaptic transistors, current research predominantly employs single device for simulating neural network training.^[^ ^40^ ^]^ Evidently, this approach lacks persuasiveness because it does not account for the mutual interference between multiple devices in an array and fails to demonstrate the practical effects of array‐based implementations.^[^ 41, 42, 43, 44, 45 ^]^ Therefore, it is crucial to utilize real EGTs arrays to showcase unsupervised learning.

In this study, a 20 × 20 EGTs array with a coplanar gate structure was fabricated using photolithography, with indium‐gallium‐zinc‐oxide (IGZO) as the semiconductor channel and polyacrylonitrile (PAN) doped with C_2_F_6_LiNO_4_S_2_ as the electrolyte. We employed EGTs as synaptic units in a hardware neural network and found that EGTs exhibit a large range of conductivity, low read–write energy consumption (6.984 fJ), excellent conductivity reproducibility, and quasilinear update characteristics, indicating their significant potential in neuromorphic computing. In addition, we verified the minimal interference between adjacent devices in the EGTs array during the programming process, confirming that our proposed EGTs array not only enables precise device programming but also virtually eliminates interference between neighboring devices. Furthermore, based on 54 transistors within the EGTs array, unsupervised learning was successfully demonstrated through experiments using a winner‐takes‐all (WTA) neural network. After 50 training iterations, the neural network successfully achieved perfect classification of test‐set letters, with an accuracy of 100%, highlighting the foundation laid by our designed EGTs array for constructing a larger‐scale unsupervised learning system.

## 2 Results and Discussion

### 2.1 The Processing of EGTs Arrays and Characterization of Electrolyte Thin Films

**Figure 1:**
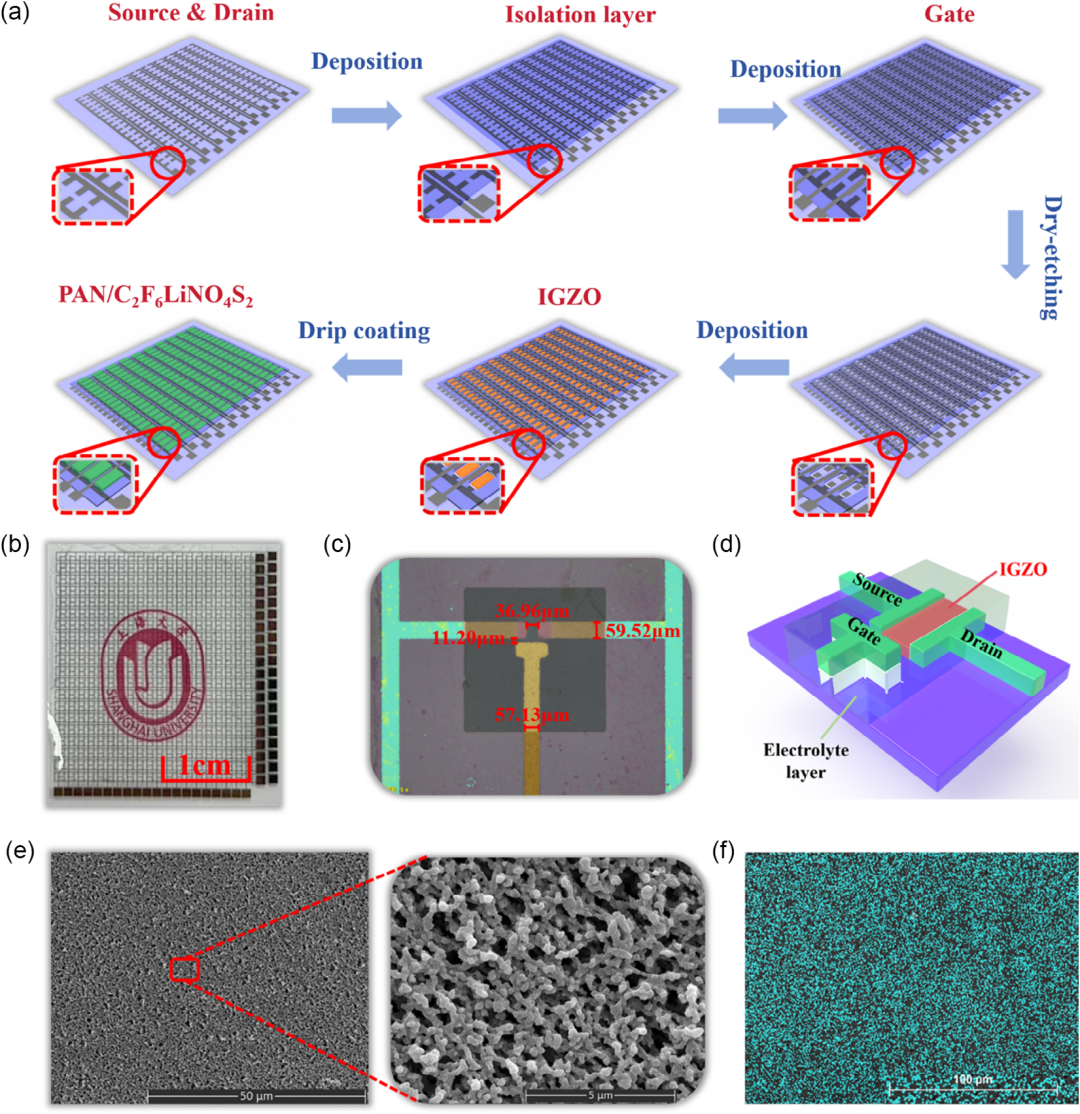
Fabrication process and physical characteristics of EGTs array and electrolyte film. a) Process diagram of the EGTs array based on photolithography. b) Photo of the prepared EGTs array. c) Picture of a single transistor in the array. d) 3D structure diagram of a single electrolyte transistor. e) SEM image of the surface of the electrolyte film (left) and a local magnified view of the film's surface (right). f) EDS analysis result for the element F (representing C_2_F_6_LiNO_4_S_2_).

The fabrication process of the EGTs array, as shown in **Figure** **1**a, is detailed in **Experimental Section**. Figure 1b shows a photograph of the array. To more clearly display the morphology of single device in the array, we also captured optical images of individual devices in the array without electrolyte coating and annotated the corresponding dimensions, as shown in Figure 1c. As a matter of fact, it is commonly acknowledged that top gate or bottom gate structure, which is used in the fabrication of conventional MOSFET, is helpful for the miniaturization of the device. However, the incompatibility between the electrolyte and the conventional Si‐based technology prevent the integration of EGTs. Therefore, we propose a coplanar structure of EGTs to accomplish this integration, as illustrated in Figure 1d. This specific structure allows the electrolyte film to be drop cast on top of the array, effectively avoiding damage to the film from other fabrication processes.^[^ 46, 47, 48, 49 ^]^ The preparation of this EGTs array based on the coplanar gate structure provides valuable experience and reference for manufacturing large‐scale arrays using similar electrolyte materials. In terms of the device's integration density, considering the stability and repeatability of the array, so far, we have only been able to achieve this level of integration density due to technical limitations. Although the density does not seem very high, we believe that higher integration densities can be achieved with improvements in process technology. Please refer to our previous work for more details.^[^ ^50^ ^]^ Here, we propose the following potential challenges for further integration. In this integration scheme, with the reduction of device size and the increase in integration density, the difficulties of interconnection and signal acquisition will intensify.^[^ ^51^ ^]^ The planar structure of a single transistor with a coplanar gate used in the EGTs array is depicted in Figure S1a, Supporting Information. This structure leverages the double‐layer effect to directly couple the gate voltage to the semiconductor channel. When an external voltage is applied at the gate, ions accumulated at the interface of PAN/C_2_F_6_LiNO_4_S_2_ electrolyte and the electrode result in a significant double‐layer capacitance and strong lateral field coupling.^[^ ^52^ ^]^ This is crucial for constructing artificial synaptic networks. In the search for the PAN/C_2_F_6_LiNO_4_S_2_ mixing ratio with the maximum areal capacitance, we characterized the film capacitance under different mixing ratios, as shown in Figure S1b, Supporting Information. From the graph, it is observed that a substantial double‐layer capacitance exists in the low‐frequency range, but as the frequency increases from 20 Hz to 2 MHz, the capacitance significantly decreases. The areal capacitance of the film varies with the doping concentration of C_2_F_6_LiNO_4_S_2_. When PAN is mixed with C_2_F_6_LiNO_4_S_2_ at a 1:1 mass ratio, the resulting electrolyte film exhibits the highest specific capacitance at a frequency of 20 Hz, reaching 0.55 μF cm^−2^. In contrast, when the mass ratio of PAN to C_2_F_6_LiNO_4_S_2_ is 2:1, the specific capacitance decreases to 0.48 μF cm^−2^; when the mass ratio is 3:1 ratio, it further decreases to 0.46 μF cm^−2^. The specific capacitance of the pure PAN electrolyte film, without C_2_F_6_LiNO_4_S_2_ doping, is only 0.33 μF cm^−2^. Furthermore, a detailed analysis was conducted on the surface of the PAN/C_2_F_6_LiNO_4_S_2_ mixed electrolyte film with a 1:1 mass ratio, and its scanning electron microscope (SEM) image is displayed in Figure 1e. The left image represents the overall morphology of the film, whereas the right SEM image is an enlarged view of the film's surface. The SEM images indicate a relatively uniform electrolyte surface with limited roughness. This result was confirmed by energy‐dispersive X‐ray spectroscopy (EDS) analysis for element F, representing C_2_F_6_LiNO_4_S_2_, as shown in Figure 1f.

### 2.2 The Electrical and Synaptic Performance of EGTs

**Figure 2:**
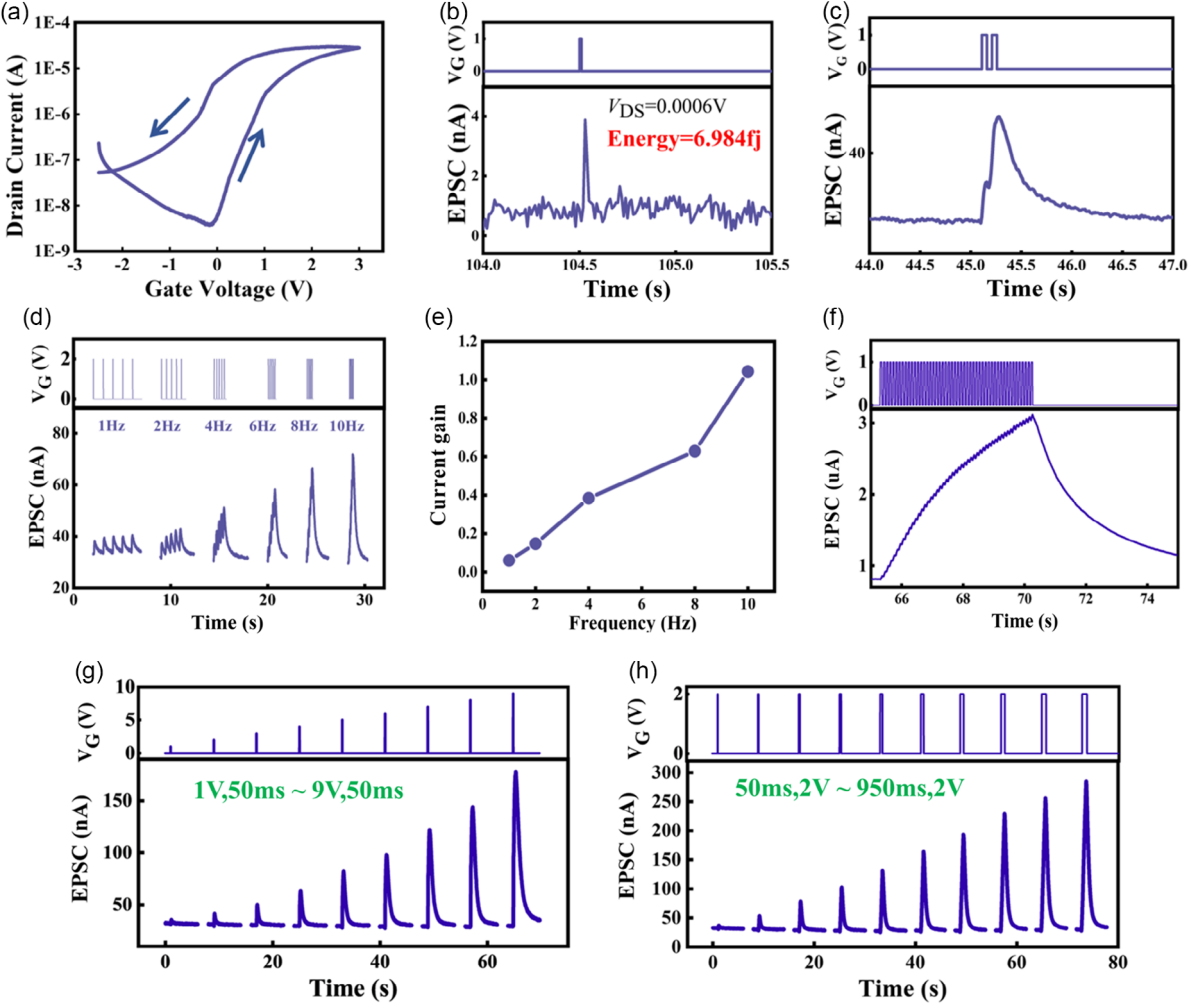
The electrical and synaptic performance of individual transistors in the EGTs array. a) Hysteresis transfer curve of individual transistors in the EGTs array within the voltage range of −2.5 to 3 V, showing a large hysteresis window indicative of significant potential for achieving synaptic characteristics. b) EPSC current triggered by a pulse voltage (*V*_G_ = 1 V, *V*_DS_ = 0.0006 V, *t*_pulse_ = 3 ms), with a calculated energy consumption of 6.984 fJ. c) EPSC triggered by a pair of presynaptic pulses (1 V, 60 ms), where the EPSC triggered by the second pulse is 45.5 nA, significantly larger than the 35.7 nA triggered by the first pulse. d) EPSC triggered by different pulse frequencies (1–10 Hz, *V*_G_ = 2 V, *V*_DS_ = 0.05 V, *t*_pulse_ = 50 ms), showing an increase in triggered EPSC with increasing frequency. e) Current gain at different frequencies, G = (A5 – A1)/A1, indicating an increase in current gain from 0.06 to 1.04 as the frequency increases from 1 to 10 Hz, suggesting significant potential for high‐pass filtering applications. f) EPSC responses to 50 consecutive pulses (*V*_G_ = 1 V, *t*_pulse_ = 50 ms). g) Changes in EPSC as the voltage amplitude gradually increases from 1 to 9 V (*V*_DS_ = 0.05 V, *t*_pulse_ = 50 ms). h) Changes in EPSC as the pulse voltage width increases from 50 to 950 ms (*V*_G_ = 2 V, *V*_DS_ = 0.05 V).

As shown in **Figure** **2**a, the transfer characteristics curve of EGTs (*V*_D_ = 0.4 V) is obtained by performing a forward sweep of *V*_G_ from −2.5 to 3 V and then returning to −2.5 V. This curve exhibits a distinct hysteresis window, which is advantageous for simulating the plasticity of synaptic performance in subsequent steps. The threshold voltage (*V*_th_) for individual transistors in the EGTs array is −0.002 V, with a subthreshold swing of 0.26 V decade^−1^. Under the conditions of *V*_DS_ = 0.0006 V, pulse signals with pulse widths and amplitudes of 3 ms and 1 V, respectively, were applied to the gate terminal, triggering the excitatory postsynaptic current (EPSC), as depicted in Figure 2b. This phenomenon occurs because when a positive voltage is applied to the gate, a forward electric field forms between the gate and the channel. This drives positively charged ions to gradually migrate to the interface between the gate dielectric and the channel, inducing channel current. As the pulse voltage is removed from the gate, the ions gradually diffuse back to their initial state, causing the EPSC to return to its initial state as well.

The maximum value of the EPSC is 3.88 nA. The individual energy consumption of a single pulse peak for a transistor in the array was calculated based on the formula (1):

(1) $$E_{\text{pre pulse}}=I_{\text{peak}}\cdot V_{\text{DS}}\cdot t$$

where *I*_peak_ and *t* represent the peak and width of the pulse, respectively, and the calculated minimum energy consumption for a single pulse of the transistor is 6.984 fJ, which is comparable with the energy consumption of a biological synapse.^[^ 30, 53, 54, 55, 56, 57 ^]^ To further investigate the synaptic transistor's paired pulse facilitation (PPF) behavior, we applied two consecutive presynaptic pulses to the gate terminal of the synaptic transistor. Each pulse had an amplitude of 1 V, a width of 60 ms, and an interval of 40 ms. As shown in Figure 2c, the EPSC triggered by the second pulse is 72.1 nA, significantly higher than the 43.6 nA triggered by the first pulse. The reason for this double‐pulse facilitation is the relatively slow ion motion rate in the solid‐state electrolyte film, which cannot respond promptly to the pulse voltage at the gate. When the subsequent pulse stimulus arrives, the ions that did not fully respond during the first pulse further contribute, resulting in an increase in the EPSC.^[^ 58, 59, 60 ^]^ Figure 2d demonstrates the simulation of the synaptic transistor's high‐pass filtering characteristics using six sets of pulse signals with different frequencies (pulse width of 50 ms, pulse amplitude of 2 V, *V*_DS_ = 0.05 V). We set the amplitude of the EPSC triggered by the first pulse as A1 and the amplitude of the EPSC triggered by the fifth set of pulses as A5. When 1 Hz frequency pulses are applied to the gate, the EPSC remains relatively unchanged. However, as the pulse frequency increases from 1 to 10 Hz, the response of the synaptic transistor gradually intensifies, as shown in Figure 2e. The gain of the EPSC ((A5–A1)/A1) increases from 0.06 to 1.04, indicating that the device can serve as a high‐pass filter in the information transmission process. This phenomenon occurs as with the increase in pulse frequency, the time intervals between presynaptic pulses shorten, leaving insufficient time for ions to return to their equilibrium positions. This results in more ions accumulating at the gate dielectric and channel surfaces, leading to larger EPSC.^[^ 57, 61, 62, 63 ^]^ Figure 2f displays the EPSC response after applying 50 presynaptic pulses (pulse width of 50 ms, *V*_G_ = 1 V, *V*_DS_ = 0.4 V) to the synaptic transistor. It is observed that the amplitude of the subsequent EPSC is higher than that of the previous one. This occurs because if a second stimulus is triggered before the residual ions fully recover, these residual ions combine with the ions triggered by the second stimulus, leading to a higher amplitude of the subsequent EPSC. This mechanism is similar to PPF. Figure 2g illustrates the effect of different voltage stimuli on the postsynaptic current (pulse width of 60 ms, *V*_DS_ = 0.05 V). As the applied voltage gradually increases from 1 to 9 V, the generated EPSC increases from 35.76 to 178 nA. This indicates that the peak of the EPSC increases with the increment of the stimulus voltage. As higher voltages are applied, a greater number of ions are induced in the electrolyte layer, resulting in an increase in the EPSC. Figure S2a, Supporting Information, displays the relationship between pulse amplitude and the corresponding EPSC size. It is evident from the graph that as the stimulus voltage increases, the generated EPSC exhibits an approximate linear relationship. This also suggests that it performs well in a programming approach involving a gradual increase in voltage amplitude.^[^ 64, 65 ^]^ By applying electrical signals of varying pulse widths (*V*_G_ = 2 V, *V*_DS_ = 0.05 V) to the gate terminal of the synaptic transistor, ranging from 50 to 950 ms, we obtained EPSC responses under different pulses width stimuli. As shown in Figure 2h, with an increase in pulse duration, the EPSC generated by the synaptic transistor also increases, and the time required for it to return to its initial state becomes longer. This is because, as the pulse duration extends, more ions accumulate at the interface between the gate dielectric layer and the active layer, resulting in the accumulation of more electrons at the channel interface and, consequently, an increase in EPSC. Figure S2b, Supporting Information, depicts the relationship between pulse width and the corresponding EPSC size. It is apparent from the graph that the increase in pulse width results in EPSC responses that approximate a linear trend, indicating its notable effectiveness in a programming approach involving a gradual increase in voltage pulse width.^[^ 64, 65 ^]^

### 2.3 Array Programming Interference Analysis and Uniformity Characterization

**Figure 3:**
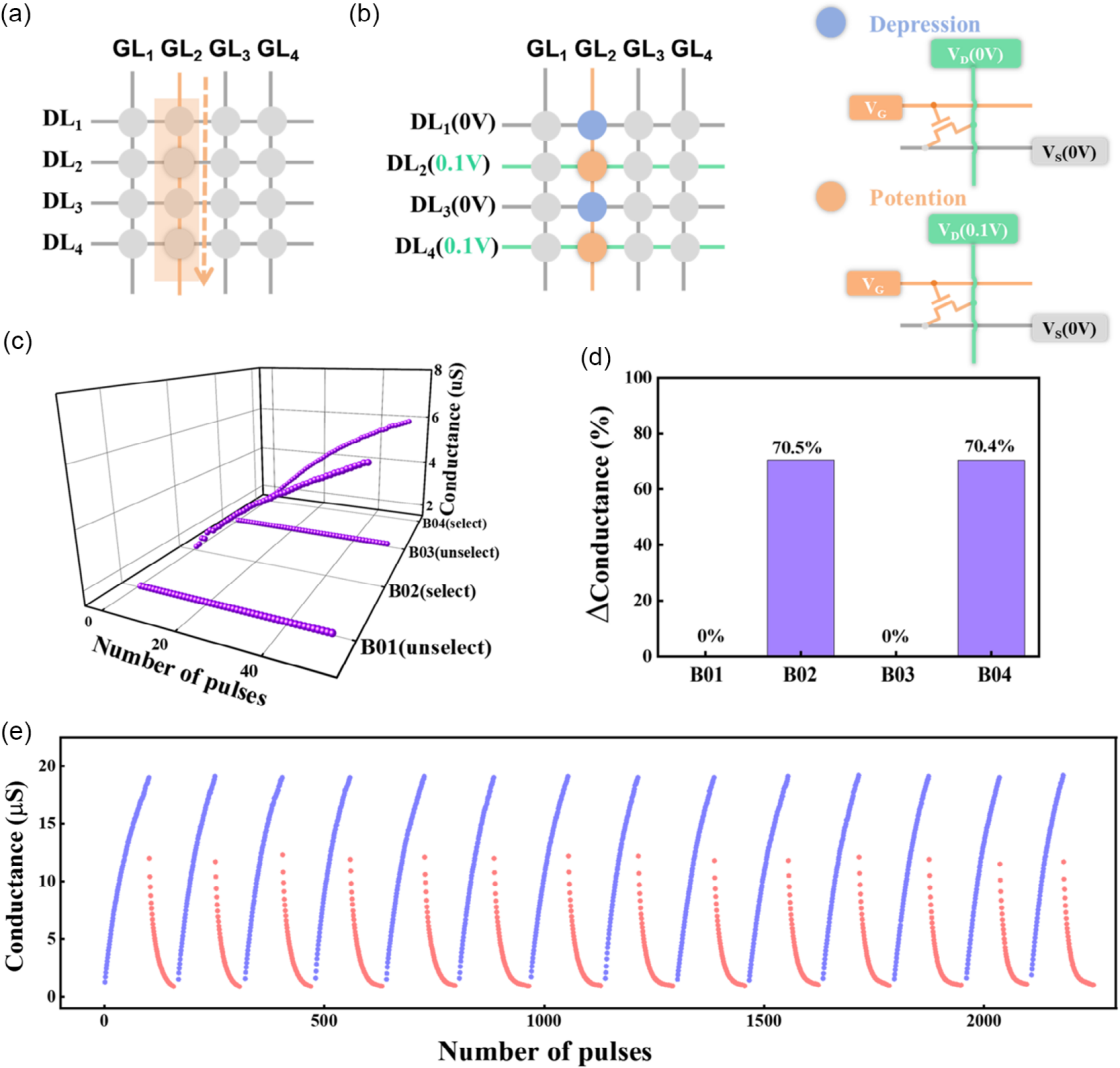
Crosstalk in the array and device durability. a) An example of a 4 × 4 array, with one gate controlling a column of devices. b) The configuration for reducing device programming interference in the EGTs array (left) and the specific arrangement of individual devices within the array (right). c) Changes in the conductance of four adjacent devices sharing a common gate under the trigger of 50 consecutive excitatory pulses (pulse voltage: 1 V, width: 60 ms). The selected device's conductance shows a significant increase, whereas the conductance of the unselected devices remains relatively unchanged. d) The electrical conductance changes for the two selected devices are 70.5% and 70.4%, whereas the unselected two devices show no electrical conductance change, registering at 0%. e) Durability characteristics of EGTs subjected to over 2200 pulses.

In the EGTs array, transistors within the same column share a common gate electrode port. Therefore, when programming a specific device within the array, other devices in the same column may also experience programming interference (**Figure** **3**a).^[^ 66, 67 ^]^ This is disadvantageous for subsequent differential computing tasks. To address this issue, we devised an independent programming method based on the EGTs array design. During the programming, a selected device receives programming pulses on its GL while setting SL to 0 V and DL to 0.1 V. For nontargeted devices, we implemented an inhibition procedure by setting their DL to a low voltage level (0 V) (Figure 3b). This reduced interference by keeping these nontargeted devices deactivated. To demonstrate the practical effect, we selected four adjacent devices within the same column, sharing a common gate electrode port. We applied a continuous sequence of 50 trigger pulses to the common gate electrode port (pulse voltage of 1 V with a duration of 60 ms). We configured devices for excitation and inhibition, as previously described. The results showed a significant increase in channel conductance for the two selected devices, whereas the other two nonselected devices maintained nearly constant channel conductance (Figure 3c). To provide a clearer representation of the change in channel conductance for these devices, we calculated the relative change in channel conductance for the four adjacent devices sharing the common gate electrode port. The relative change in channel conductance for B01, B02, B03, and B04 was 0%, 70.5%, 0%, and 70.4%, respectively (Figure 3d). This indicates that this programming method is highly effective for achieving independent programming. Furthermore, this pulse programming method allows for individual programming of devices within the array that have different conductance states. To evaluate the durability of EGTs, more than 2200 P–D pulses (*V*_P_ = 2 V, *V*_D_ = ‐2 V, *t*_pulse_ = 50 ms) were applied to the EGTs. As shown in Figure 3e, even after the application of over 2200 pulses, EGTs devices remained stable and exhibited excellent repeatability compared with the initial measurements.

To achieve CIM in EGTs arrays, small differences between devices are necessary. To assess the mutual differences between devices, we characterized the electrical characteristics of 100 devices in the array. By performing transfer characteristics curve measurements on 100 transistors selected from a 20 × 20 array of synaptic transistors, *V*th and SS were extracted. Figure S3a, Supporting Information, displays the distribution of *V*th for these 100 devices, ranging from −0.8 to 0.6 V, the average of the *V*th for the selected 100 devices is 0.19 V, with a standard deviation of 0.22 V. Figure S3b, Supporting Information, shows the distribution of SS for these 100 devices, which generally falls within the range of 0.1–0.9 V decade^−1^, the average of the SS for the selected 100 devices is 0.51 V decade^−1^, with a standard deviation of 0.14 V decade^−1^. Using the number of pulses to represent the learning iterations, 50 pulses (with a pulse width of 60 ms, pulse amplitude of 1 V, and *V*_DS_ = 0.4 V) were administered at various locations within the array. The corresponding EPSC of each device was measured at 1, 5, 10, 20, 30, and 50 pulses after stimulation. The varying color intensity observed in the grid cells of the figure indicates the strength of the EPSC generated by the corresponding unit in the array. The grid cells in the figure exhibit varying color intensity, reflecting the magnitude of EPSC generated by each unit in the array. With the increase in the number of learning iterations, the memory level showed improvement, as illustrated in Figure S3c, Supporting Information. The synaptic transistor array successfully demonstrated dynamic memory, suggesting that it has promising potential for practical applications in image processing.

Figure S3d, Supporting Information, shows the channel conductance variation of a single transistor in the array under a single‐period pulse stimulation, with linearity values of 1.16 for the potentiation phase and 3.29 for the depression phase, demonstrating acceptable linearity.^[^ 68, 69 ^]^ A two‐layer perceptron is a basic neural network model consisting of 400 input neurons, 100 hidden neurons, and 10 output neurons, as shown in Figure S3e, Supporting Information. Through repeated forward propagation and backpropagation processes, the two‐layer perceptron can gradually learn the weights that adapt to the input data, enabling classification or prediction of the input data. Figure S3f, Supporting Information, displays the accuracy of the transistor over 125 training cycles, with a significant improvement in perceptron accuracy in the first 10 training cycles. Throughout the entire training process, the highest accuracy achieved is 92.02%.

### 2.4 Implementing Unsupervised Learning Functionality Based on EGTs Array

**Figure 4:**
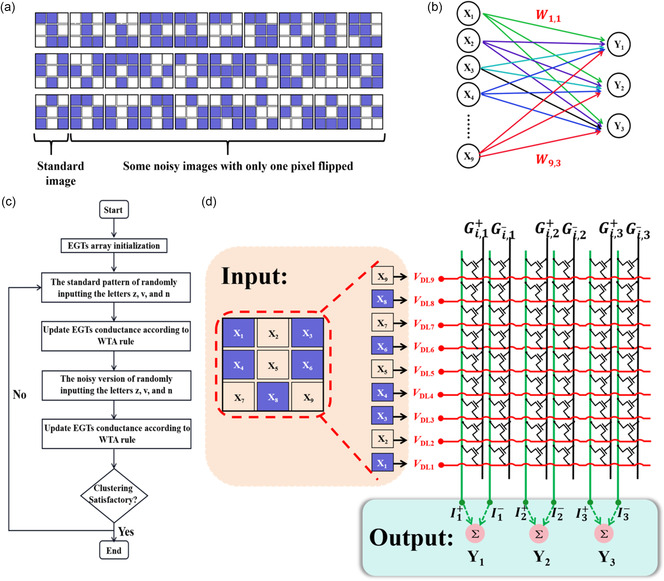
The architecture diagram for unsupervised learning. a) Standard images of letters “z”, “v”, and “n” and their noisy versions with only one pixel flipping. b) Architecture of the neural network. c) Flowchart for WTA network training based on EGTs array. d) Training process of the letter“v” as an example within the neural network system.

The EGTs array was configured as a WTA neural network, using EGTs as analog synapses, for the classification of standard letters z, v, n, and their noisy versions (**Figure** **4**a). This network was trained based on the actual EGTs array, meaning that no external computer simulation models were used during both the training and inference processes. The network's input layer consists of nine neurons, each corresponding to one of the nine pixels in the pattern, whereas the output layer is composed of three neurons, each corresponding to one of the three output categories (Figure 4b). The network's output results *Y*_*j*_ are primarily determined by formula (2):

(2) $$\begin{array}{c} Y_{i}=\sum_{i=1}^{9}x_{i}W_{i,j}\\ \sum_{i=1}^{9}x_{i}(G_{\left(i,j\right)}^{+}-G_{i,j}^{-}) \end{array}$$

where $x_{i}\;$ is the input voltage for the ith pixel, where dark pixels represent high‐level input voltage and light pixels represent low‐level input voltage. Here, $W_{i,j}$ represents the synaptic weight, which is equal to the difference in channel conductance between two adjacent devices ($G_{\left(i,j\right)}^{+}-G_{i,j}^{-}$). Using this differential subtraction method to represent synaptic weights is effective in achieving both positive and negative weight values and can effectively increase the dynamic range of synaptic weights. Furthermore, representing synaptic weights using the differential subtraction of channel conductance between adjacent devices assists in reducing device‐to‐device variability, as adjacent devices exhibit highly similar characteristics.^[^ 32, 70 ^]^

The training process for unsupervised learning is illustrated in Figure 4c. Initially, the EGTs array needs to undergo an initialization step to ensure that the devices within the array are in their initial state. Next, we randomly select one of the standard letters “z”, “v”, or “n” as an input image, with each image representing one training epoch. The training phase involves using the standard patterns of the letters z, v, and n, as well as their noisy versions. Train until the synaptic weights of each neuron are sufficiently updated. In the process of conducting unsupervised learning, a pulse width of 50 ms and a pulse amplitude of 1 V were applied to the gate, whereas a drain voltage of 0.4 V was used. Taking the standard letter “v” as an example, the training process is as follows (see Figure 4d): First, the image of the standard letter “v” is decomposed into nine pixels, and these pixels are fed into the neural network. Subsequently, following the WTA rule, synaptic weights within the neural network undergo updates. The specific weight update process is as follows: after inputting the standard “v” letter's pattern into the neural network model, the network generates three output results. These three output results are then compared, and the output neuron corresponding to the maximum output value is declared as the winner. Only the synaptic weights connected to the winning neuron are updated, whereas other synaptic weights remain unchanged. Synaptic weights connected to the winning neuron and associated with high‐level input are strengthened. Conversely, synaptic weights connected to the winning neuron and associated with low‐level input are suppressed. For example, if, after inputting the standard letter “v,” neuron Y_1_ produces the highest output value, Y_1_ is declared as the winner. In this case, synaptic weights connected to Y_1_, such as $W_{1,1},W_{3,1},W_{4,1},W_{6,1},\text{and }W_{8,1}$, would be increased ($G_{1,1}^{+},G_{3,1}^{+},G_{4,1}^{+},G_{6,1}^{+},\text{and }G_{8,1}^{+}$ increased and $G_{1,1}^{-},G_{3,1}^{-},G_{4,1}^{-},G_{6,1}^{-},\text{ and }G_{8,1}^{-}$ unchanged), whereas synaptic weights connected to *Y*_1_, such as $W_{2,1}$, $W_{5,1}$, $W_{7,1}$, and $W_{9,1}$, would be suppressed ($G_{2,1}^{+}$, $G_{5,1}^{+}$, $G_{7,1}^{+}$, $\text{and }G_{9,1}^{+}$ unchanged, $G_{2,1}^{-}$, $G_{5,1}^{-}$, $G_{7,1}^{-}$, $\text{ and }G_{9,1}^{-}$ increased). After a sufficient number of training iterations, a test set consisting of both the standard and noisy versions of these letters is introduced into the trained neural network model to evaluate its clustering performance with respect to these letters.

**Figure 5:**
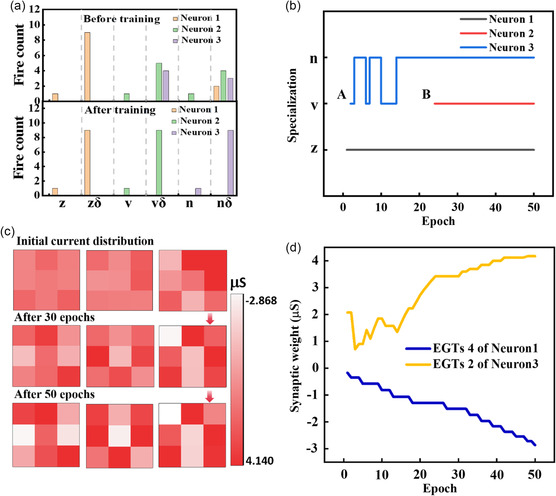
Presentation of unsupervised learning results. a) Classification results of the test set by the neural network system before and after training. Many patterns were misclassified before training, but after training, it effectively classifies standard letters and noisy letters in the test set. b) Changes in the different letters represented by the output neurons during the training process. c) Evolution of synaptic weights of the three output neurons during 50 rounds of training; as training progresses, the shapes represented by each output neuron become gradually clearer. d) Example of the synaptic weight update process for the fourth synapse of the first neuron and the second synapse of the third neuron.

**Figure** **5**a illustrates the clustering results for letters in the EGTs array before and after training. It can be observed that before training, many patterns are misclassified, as shown in the upper part of Figure 5a. At this point, the network does not yet possess the capability to effectively cluster letters. However, after multiple rounds of training with the standard and noisy patterns of the letters z, v, and n, the EGTs array gains the ability to successfully cluster the letters, with an accuracy of 100%, as depicted in the lower part of Figure 5a. To better understand the convergence behavior of the algorithm, we define a specialization function Si for each neuron *i*, representing the pattern *x* (z, v, n) for which the neuron produces the maximum output yi. During the training of the neural network, when each neuron uniquely corresponds to different letter patterns and remains unchanged, we can consider the network's classification a success. Figure 5b shows the changes in the output neurons representing different letters during the training process. From the figure, it can be seen that after 24 training cycles, the neural network achieves successful classification. Subsequently, neurons 1, 2, and 3 correspond to patterns z, v, and n, respectively. Between points A and B, neuron 3 points to both letters *v* and *n*. This is due to the initial weight distribution of the neural network system not creating a significant distinction between the patterns *v* and *n*, leading to an illusion of misclassification. However, with the increasing number of training cycles, the synaptic weights of the neural network receive updates. When confronted with the input of v and n once again, the system successfully classifies them. Figure 5c demonstrates the evolution of 27 synaptic weights during the neural network training process. Before any training, the weight distribution chart shows no apparent patterns. With an increasing number of training cycles, the distribution chart representing the letter patterns in the synaptic weights gradually becomes clearer. After ≈30 training cycles, neurons 1, 2, and 3 have exhibited relatively clear letter patterns, corresponding to the letters z, v, and n. To better illustrate this process, we use the fourth transistor of neuron 1 and the second transistor of neuron 3 as examples, detailing the evolution of their weights, as shown in Figure 5d. They start from similar initial values and then evolve according to the WTA rule. After multiple training iterations, they eventually form two distinct states. The WTA algorithm demonstrated in this work can also be extended to multilayer WTA networks to address more complex input features.

## 3 Conclusions

In summary, we fabricated a 20 × 20 synaptic transistor array utilizing photolithography, with IGZO as the semiconductor channel and PAN doped with C_2_F_6_LiNO_4_S_2_ as the electrolyte. The experiments have demonstrated that individual synaptic transistors within the EGTs hold significant potential in neuromorphic computing. In addition, we verified the minimal interference between adjacent devices in the EGTs array during the programming process, affirming that our proposed EGTs array not only facilitates precise device programming but also virtually eliminates interference between neighboring devices. Based on 54 transistors within the EGTs array, unsupervised learning was successfully demonstrated through experiments using a WTA neural network. After 50 training iterations, the neural network successfully achieved perfect classification of test‐set letters, with an accuracy of 100%. This implies that our designed EGTs array can be used to construct a larger unsupervised learning system. The work demonstrates the potential of EGTs for constructing large‐scale integration synaptic array toward efficient computing architectures.

## 4 Experimental Section

4.1

4.1.1

##### Film Preparation

At room temperature, 3 mL of dimethylformamide solution was added to a clean sample bottle and then sequentially 0.15 g of PAN powder (purchased from Sinopharm Chemical Reagent Co., Ltd.) and a magnetic stir bar were added. The mixture was stirred with a magnetic stirrer at a speed of 500 r min^−1^ for 2 h, then 0.15 g of C_2_F_6_LiNO_4_S_2_ powder was added (purchased from Beijing InnoChem Science & Technology Co., Ltd.), and stirring was continued for ≈12 h to obtain a mixed solution of PAN and C_2_F_6_LiNO_4_S_2_ with a mass ratio of 1:1.

##### Array Fabrication

1) Using physical vapor deposition equipment, 150 nm thick Mo and 50 nm thick ITO were sputtered onto the cleaned glass substrates. Photolithography and wet etching processes were used to pattern the metal electrode layer to create source‐drain electrodes; 2) Plasma‐enhanced chemical vapor deposition was employed to deposit a 250 nm thick SiO_2_ layer above the source‐drain electrodes as an isolation layer; 3) Once again using physical vapor deposition equipment, 150 nm thick Mo and 50 nm thick ITO were sputtered onto the isolation layer and patterned to form gate electrodes; 4) Photolithography and dry etching were used to pattern the isolation layer, defining the regions where the array's electrodes and gate electrodes interact with the active layer; 5) Once again using physical vapor deposition equipment, a 30 nm thick layer of IGZO was sputtered onto the substrate and patterned to create the channels of EGTs; and 6) The prepared PAN/C_2_F_6_LiNO_4_S_2_ mixed solution was drop coated onto the array and left to stand in the air for half an hour. After the liquid solution solidified, it was ready for testing.

##### Array Measurement

At room temperature, the electrical properties of EGTs array were measured using a semiconductor analyzer (Keithley 4200A‐SCS). The synaptic characteristics of the EGTs array were measured using a test system consisting of a semiconductor analyzer (Keithley 4200A‐SCS) and a signal generator (Rigol DG822).

## Conflict of Interest

The authors declare no conflict of interest.

## Supporting information

Supplementary Material

## Data Availability

The data that support the findings of this study are available from the corresponding author upon reasonable request.
