# Supplementary material for: Electrolyte‐Gated Transistor Array (20 × 20) with Low‐Programming Interference Based on Coplanar Gate Structure for Unsupervised Learning
Source: Small Sci. 2024 Mar 3;4(4):2300306. doi: 10.1002/smsc.202300306 (PMC11935182; doi:10.1002/smsc.202300306)
Supplement: Supplementary file 1 — Supplementary Material [file SMSC-4-2300306-s001.pdf]

Supporting Information

Title: Electrolyte-gated transistor array (20×20) with low-programming interference based on coplanar gate structure for unsupervised learning

Wenkui Zhang[a], *Jun Li*[a,b,*], *Mengjiao Li*[a], *Yi Li*[a], *Hong Lian*[b], *Wenqing Gao*[a], *Benxiao Sun*[a], *Fei Wang*[a], *Lian Cheng*[a], *Hanqi Yu*[a], *Lianghao Chen*[a], *Jianhua Zhang*[a,b,*]

[a] School of Microelectronics, Shanghai University, Shanghai 201800, China.

[b] MOE Key Laboratory of Advanced Display and System Applications, Ministry of Education, Shanghai University, Shanghai 200072, China

* Corresponding author.

E-*mail address*: lijun_yt@shu.edu.cn (J. Li), jhzhang@oa.shu.edu.cn (J.H.Zhang).

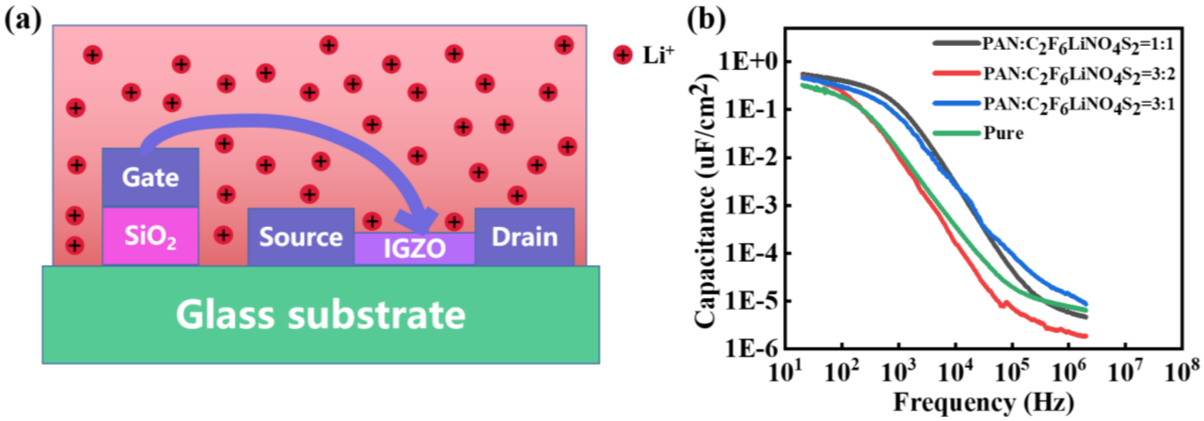

Figure S1. (a) The planar diagram of the coplanar gate structure. (**b**) The capacitance variation curve encompasses the changes in capacitance of PAN mixed with $C_2F_6LiNO_4S_2$ at different ratios (1:1, 2:1, 3:1) as well as pure PAN, within the frequency range of 20Hz to 2MHz.

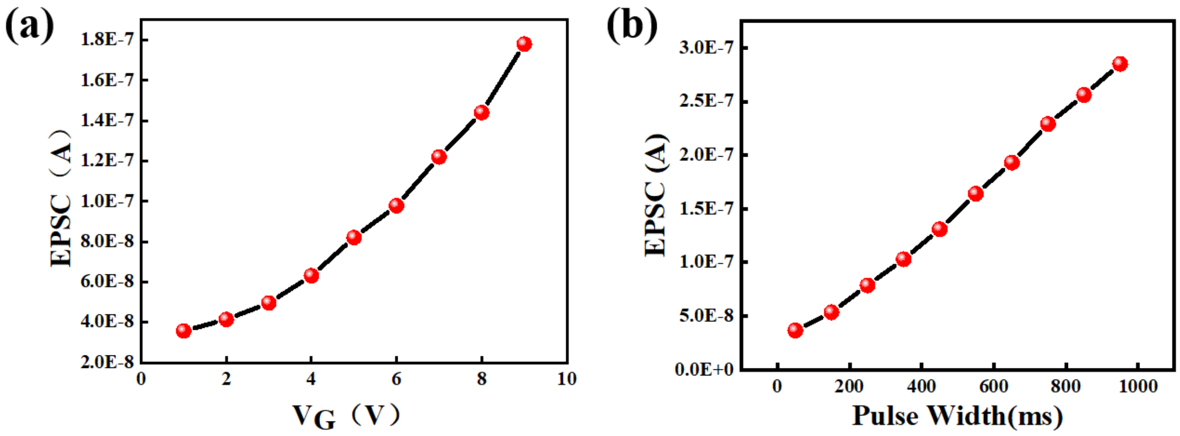

Figure S2. (a) The relationship between pulse amplitude and the corresponding EPSC magnitude. **(b)**The relationship between pulse width and the corresponding EPSC magnitude.

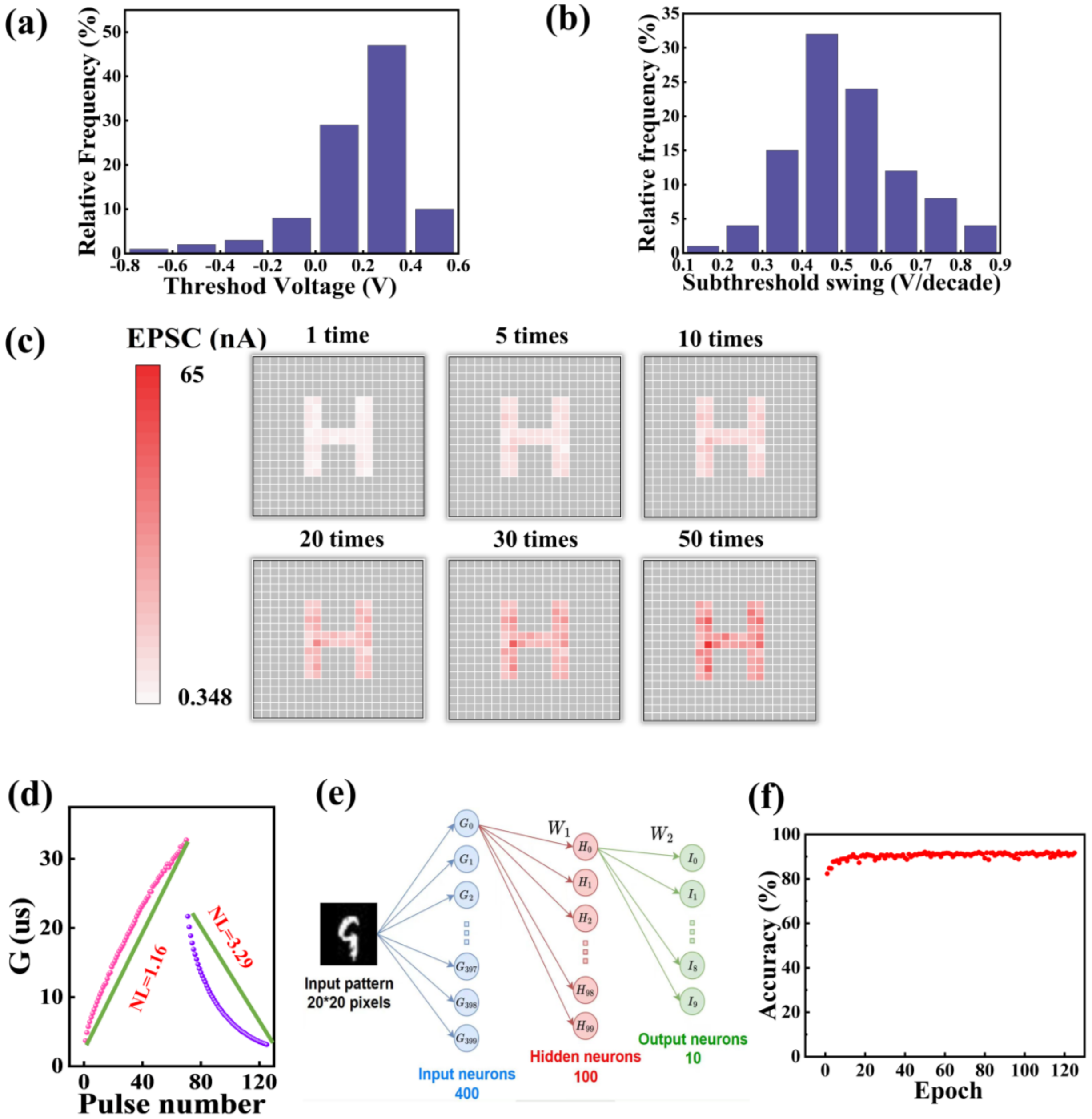

Figure S3. (a) Threshold Voltage Distribution Percentage. (b) Subthreshold Swing Distribution Percentage. (c) Dynamic learning graph of digits. (d)Changes in device channel conductance during a single-cycle pulse, with linearity values of 1.16 for the excitation process and 3.29 for the inhibition process. (e)The schematic diagram of a two-layer perceptron. (f)Perception accuracy of an artificial neural recognition system based on a parameter set of single transistors in an EGTs array.
